# A Systematic Review and Meta‐Analysis Comparing the Diagnostic Accuracy of Swab Cultures and Tissue Biopsy for Diagnosing Diabetic Foot Infections

**DOI:** 10.1155/ije/6364693

**Published:** 2026-08-20

**Authors:** Roberto S. Heck, ⁠Rodrigo S. Lins, ⁠Daniela R. Speransa, Pedro Lopez, ⁠Lessandra Michelin

**Affiliations:** ^1^ Programa de Pós-Graduação em Ciências da Saúde, Universidade de Caxias do Sul, Caxias do Sul, Rio Grande do Sul, Brazil, ucs.br; ^2^ Sociedade de Infectologia do Estado do Rio de Janeiro, Rio de Janeiro, Brazil; ^3^ Faculdade de Medicina, Instituto de Educação Médica (IDOMED), Universidade Estácio de Sá, Angra dos Reis, Brazil, estacio.br; ^4^ Unidade de Terapia Transfusional, Serviço de Hemoterapia, Hospital de Clínicas de Porto Alegre, Porto Alegre, Rio Grande do Sul, Brazil, hcpa.edu.br; ^5^ Centre for Innovative Pleural Research, Institute for Respiratory Health, Nedlands, Western Australia, Australia; ^6^ School of Medical and Health Sciences, Edith Cowan University, Joondalup, Western Australia, Australia, ecu.edu.au; ^7^ Grupo de Pesquisa Em Exercício Para Populações Clínicas (GPCLIN), Universidade de Caxias do Sul, Caxias do Sul, Rio Grande do Sul, Brazil, ucs.br

**Keywords:** biopsy, diabetic foot, diagnostic accuracy, infection, swab

## Abstract

**Objectives:**

To evaluate and compare the diagnostic accuracy and concordance of swab cultures and tissue biopsy for identifying causative pathogens. Furthermore, to compare the sensitivity, specificity and overall diagnostic performance of swab cultures and tissue biopsies, using tissue biopsy as the reference standard in infected diabetic foot wounds.

**Methods:**

We conducted a systematic review and meta‐analysis using CINAHL, Embase and PubMed for published studies from inception to April 2025. Eligible studies compared the diagnostic accuracy or the list of pathogens of swab cultures and tissue biopsy in adult patients diagnosed with diabetic foot infections. A random‐effects diagnostic accuracy meta‐analysis was undertaken.

**Results:**

Seventeen studies were included. Swab cultures identified 765 Gram‐positive microbial pathogens compared to 875 identified by tissue biopsies and 457 Gram‐negative pathogens compared to 452 identified by tissue biopsies. The three most prevalent microbial species were *Staphylococcus aureus*, *Streptococcus* spp. and *Enterococcus* spp. Pooled sensitivity and specificity of swab cultures for detecting infected diabetic foot wounds were 84.8% and 68.0%, respectively, using tissue biopsy as the reference standard. Subgroup analyses indicated better pooled sensitivity and specificity for Gram‐negative (85.2% and 92.9%, respectively) compared to Gram‐positive pathogens (76.8% and 81.0%, respectively).

**Discussion:**

Our findings are that swab cultures could identify Gram‐negative pathogens and may still be acceptable for detecting Gram‐positive and overall infections. While tissue biopsy remains the reference standard, swab cultures may represent a practical and accessible alternative in situations where tissue biopsy is not feasible or not performed.

**Key Points**
• Despite the widespread use of swab cultures, this method is often undervalued and neglected.
• Swab cultures have often been disregarded and considered the least reliable method for pathogen identification.
• Swab cultures offer diagnostic performance, particularly for identifying Gram‐negative diabetic foot infections.
• While tissue biopsy remains the gold standard, swab cultures may serve as a practical and accessible alternative in resource‐limited clinical settings or when a viable wound tissue suitable for culture is too invasive.

## 1. Introduction

Diabetic foot complications are common among patients with diabetes, with a lifetime incidence as high as 25% [1, 2]. These are characterised by ulcers, infections and deep tissue damage, which are associated with reduced quality of life, increased morbidity and can ultimately result in nontraumatic lower limb amputations and mortality [1, 3–5]. Specifically, diabetic foot ulcers are particularly concerning as they are associated with longer hospital stay compared to any other diabetes‐related complications [6]. Infections are present in 58% to 82% of cases when such ulcers fail to heal, representing a major clinical burden [7]. Therefore, reliable, cost‐effective and timely identification of diabetic foot infections is critical to guide appropriate antimicrobial therapy and improve clinical outcomes.

Tissue biopsy is widely considered the gold standard for microbiological assessment of infected diabetic foot wounds [8–10]. Methods such as curettage, punch biopsy or needle aspirate of purulent secretions are accurate [11] but can be invasive, costly and technically demanding. Tissue biopsy procedures required trained healthcare professionals, sterile conditions and appropriate equipment, which can limit their availability, especially in resource‐limited settings. Moreover, biopsy may be contraindicated in cases of ischaemic tissue or superficial infections due to risks such as delayed healing or further tissue damage.

So far, alternatives like swab cultures have often been disregarded and considered the least reliable method for pathogen identification [8–10], even though widely used in clinical practice due to their accessibility, noninvasiveness and relatively fast results. Regarding the methodological rigour and standardisation of swab collection, earlier approaches, such as the Z‐track technique, are no longer recommended, particularly following guideline updates [12–14] after 2011 [15]. In contrast, the Levine technique (i.e., based on rotating the swab over a defined area of viable tissue with sufficient pressure) has been increasingly endorsed by clinical guidelines and supported by empirical evidence as a more reliable method [12–14], with improved concordance to tissue biopsy when performed correctly. Concerns regarding swab cultures are related to the high number of colonisers rather than true pathogens, contamination and potential lack of concordance with tissue microbiology, which has contributed to persistent clinical scepticism [16]. Additionally, swab collection techniques remain inconsistently applied in clinical studies, with limited standardisation across settings.

Two previous systematic reviews [17, 18] attempted to compare swab cultures and tissue biopsy for diagnosing diabetic foot infections, but both concluded that the available evidence was weak to support clinical recommendations. Nearly two decades later, a comprehensive reassessment of the literature available is warranted to provide updated, evidence‐based guidance for clinicians involved in the management of diabetic foot infections. Therefore, the present systematic review and meta‐analysis aimed to (i) evaluate and compare the diagnostic accuracy of swab cultures and tissue biopsy for identifying causative pathogens, (ii) assess the concordance between microbiological results obtained from swab cultures and tissue biopsy and (iii) compare the sensitivity, specificity and overall diagnostic performance of swab cultures and tissue biopsies, using tissue biopsy as the reference standard in infected diabetic foot wounds. This study can help in guiding clinical decision‐making and identify areas where further research is needed to improve diagnostic strategies in diabetic foot care.

## 2. Methods

All procedures undertaken in the present study followed the Preferred Reporting Items for Systematic Reviews and Meta‐Analyses (PRISMA) statement [19, 20] and the Cochrane Back Review Group (CBRG) [21], with PROSPERO registration under the identifier CRD420251026179.

### 2.1. Eligibility Criteria

Studies were included in this systematic review if meeting the following criteria regarding participants, intervention, comparator, outcomes and study design (PICOS): (i) studies involving adult patients (≥ 18 years) diagnosed with diabetic foot infections as defined by the authors; (ii) studies undertaking swab culture techniques to collect microbiological specimens from foot ulcers as the index test; (iii) studies undertaking tissue biopsy, tissue curettage or deep tissue aspirate as the reference standard for diagnosing infection or identifying pathogens; (iv) studies reporting diagnostic accuracy outcomes such as sensitivity, specificity, positive predictive value (PPV), negative predictive value (NPV), concordance rates or pathogens and (v) studies reporting 2 × 2 contingency tables (true positives [TP], false positives [FP], false negatives [FN], true negatives [TN]), sufficient diagnostic performance data or the list of pathogens.

The exclusion criteria were as follows: (i) studies involving only patients with diabetic foot osteomyelitis without assessment of soft tissue infection; (ii) studies undertaking only swab cultures or tissue biopsy, curettage or aspirate; (iii) studies not reporting sufficient data to estimate sensitivity, specificity, PPV or NPV; or the number of pathogens identified through swab cultures and tissue biopsy; and (iv) studies published in a language other than English.

### 2.2. Search Strategy and Study Selection Procedure

A systematic search was conducted by one researcher using the *Cumulative Index to Nursing and Allied Health Literature* (CINAHL), Embase (via Ovid) and PubMed for published studies from inception to 9 April 2025. The search strategy was undertaken using controlled vocabulary and free‐text terms as presented in Supporting Appendix S1. A manual search of references in selected studies (i.e., backward citation chasing) was performed to detect additional eligible articles for inclusion. Titles and abstracts were first independently evaluated following the eligibility criteria during the screening phase. Eligibility was assessed independently in duplicate, with differences resolved by consensus, when disagreements occurred. In case of abstracts that did not provide sufficient information, they were selected for full‐text evaluation. Full‐text articles meeting criteria were retrieved and read independently by both reviewers and assessed for inclusion in the study.

### 2.3. Data Extraction

Data extraction was performed via a standardised form. Extracted variables included sample size, mean age, percentage of male participants, duration of diabetes, Wagner grade classification and number of samples collected. Details regarding swab collection methodology (e.g., pre‐ vs. post‐debridement, Levine or alternative techniques) were extracted to explore potential sources of variability in diagnostic performance. Additionally, data on the number and type of pathogens isolated were recorded, along with TP, FP, FN, TN and/or diagnostic accuracy outcomes such as sensitivity, specificity, PPV, NPV and concordance rates, when available. For studies that did not report diagnostic accuracy outcomes directly, these measures were calculated using the Review Manager (RevMan) software (Version 5.4; The Nordic Cochrane Centre, Copenhagen, Denmark).

### 2.4. Risk of Bias Assessment

The risk of bias was evaluated according to the Quality Assessment of Diagnostic Accuracy Studies‐2 (QUADAS‐2) tool [22]. Studies were classified as having low, high or unclear risk of bias for each domain according to the QUADAS‐2 guidelines [22]. The study risk of bias assessment for all included studies was performed by one reviewer, and the results were checked by a second reviewer.

### 2.5. Data Analysis

A descriptive and narrative synthesis of study findings was first undertaken to summarize the characteristics of the included studies, including sample size, patient demographics, infection severity, swab and biopsy techniques. Additionally, all the microbial species identified in diabetic foot infections were summarised.

The diagnostic accuracy of swab cultures compared to tissue biopsy in infected diabetic foot wounds was synthesised using a random‐effects model. The meta‐analysis was conducted using Review Manager (RevMan, Version 5.4; The Nordic Cochrane Centre, Copenhagen, Denmark), the *mada* [23, 24] and *meta* [25] packages in *R* (R Core Team, Version 4.0.3., 2020). A bivariate model, through the Reitsma approach [26], was used for modelling sensitivity and specificity while accounting for their correlation. Pooled estimates of sensitivity, specificity, FP rate and diagnostic odds ratio (DOR) were calculated, along with 95% confidence intervals (CIs). An area under the curve (AUC) and partial AUC were also estimated to summarize the overall diagnostic performance. The pooled sensitivity and specificity along with corresponding 95% CIs as well as the summary receiver operating characteristic (SROC) curve were estimated and constructed in RevMan, respectively. Between‐study heterogeneity was quantified using the *I* ^2^ statistic adapted for diagnostic test accuracy studies through the Zhou and Dendukuri approach [27], with values greater than 50% in *I* ^2^ considered indicative of high heterogeneity. Variance components (*τ* ^2^) and the correlation between sensitivity and specificity were estimated using restricted maximum likelihood (REML). A subgroup analysis was provided for Gram‐positive and Gram‐negative pathogens. Potential publication bias was not assessed given the small number of studies (*n* < 10) included in the bivariate diagnostic random‐effects meta‐analysis.

## 3. Results

**FIGURE 1:**
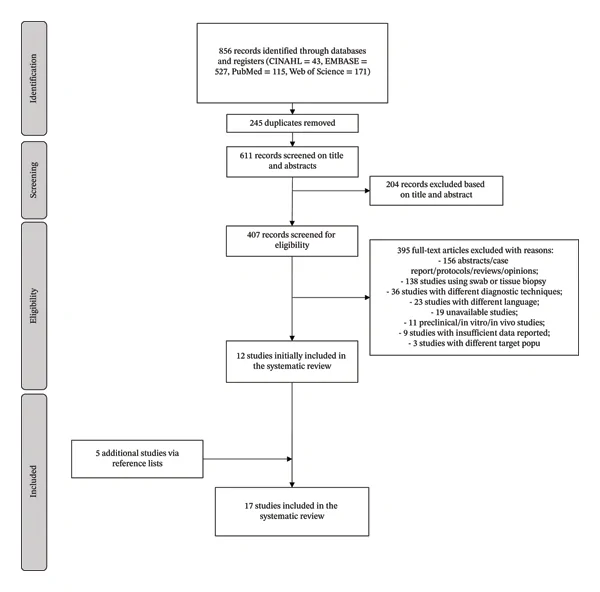
Flowchart of study selection.

A total of 856 records were identified through database searches. After removing duplicates, 611 records were screened based on title and abstract. Of these, 204 records were excluded as irrelevant to the research question, resulting in 407 records eligible for full‐text review. Records were mainly excluded because they were conference abstract/case report/thesis/dissertation/protocol/opinion/review (*n* = 156) articles, followed by the assessment of swab or tissue biopsy (*n* = 138), different diagnostic techniques (*n* = 36), different language (*n* = 23), unavailable information (*n* = 19), preclinical/in vitro/in vivo (*n* = 11), insufficient data reported (*n* = 9) and different target population (*n* = 3). While additional five studies [28–32] were identified through reference lists, a total of 17 studies [28–44] were included in this systematic review and meta‐analysis. The selection process is illustrated in Figure 1.

### 3.1. Characteristics of Study, Participant and Techniques

Most studies (76.5%) were published between 2010 and 2024. The most frequent study locations were India (29.4%) and China (11.8%). A total of 1581 participants (937 males and 644 females) with diabetic foot ulcers were included, with a median age of 62.5 (IQR: 60.3–64.1) years. Regarding ulcer severity, 29.2% of the participants presented with at least Wagner Grade III ulcers (i.e., deep ulcer associated with abscess formation, osteomyelitis or joint sepsis). The median duration of diabetes among participants was 15.5 (IQR: 12.0–17.5) years. Antibiotics were reported by 10 studies [29, 32, 33, 35–37, 40–43], with 51.8% of the participants exposed to some prior antibiotic treatment.

Regarding the microbiological assessment of diabetic foot infections, both pre‐ and post‐debridement swab collection techniques were equally undertaken in 47.1% of the cases. Among these, the post‐debridement Levine technique was used in 29.4%, while the pre‐debridement Z‐technique was used in 17.6%. The median number of isolates identified per participant via swab sampling was 1.3 (IQR: 1.1–2.2). Tissue biopsy specimens were predominantly obtained through post‐debridement (70.6%), with punch biopsies comprising 29.4% of all biopsy techniques. A median of 1.4 (IQR: 1.1–2.0) isolates per participant was identified through tissue biopsy. Risk of bias is presented in Supporting Table S1.

### 3.2. Microbial Species Identified in Diabetic Foot Infections

Information on Gram‐positive and Gram‐negative pathogens was provided by 14 studies [28–33, 35, 36, 38–41, 43, 44]. Swab cultures identified 765 Gram‐positive microbial pathogens compared to 875 identified by tissue biopsies, and 457 Gram‐negative pathogens compared to 452 identified by tissue biopsies. Regarding aerobic organisms, 1269 pathogens (93.0%) were identified by swab cultures and 1374 (91.1%) by tissue biopsies, while 95 anaerobic organisms (7.0%) were identified by swab cultures and 135 (8.9%) by tissue biopsies. Four studies reported the number of polymicrobial isolates [30, 32, 36, 40], with 72 identified by swab cultures and 69 by tissue biopsies. Sufficient information on microbial pathogens identified in diabetic foot infections was reported across 15 studies [28–36, 38–41, 43, 44]. The three most prevalent microbial species were *Staphylococcus aureus* (swab, *n* = 307 [12.0%], tissue biopsy, *n* = 327 [12.8%]), *Streptococcus* spp (swab, *n* = 132 [5.2%], tissue biopsy, *n* = 128 [5.0%]) and *Enterococcus* spp (swab, *n* = 107 [4.2%], tissue biopsy, *n* = 135 [5.3%]), followed by *Pseudomonas* spp (swab, *n* = 114 [4.5%], tissue biopsy, *n* = 120 [4.7%]).

Six studies [28, 36, 37, 40, 41, 43] assessed the concordance between swab and tissue biopsy microbial species, with substantial variability observed in metrics and results across studies. The concordance rate ranged from 25% to 90%. Williams et al. [28] found a high concordance of 90.0% between swab and tissue biopsy when bone was not exposed, but a significant reduction to 65.0% when bone exposure was present. Mutluoglu et al. [36] reported an overall concordance of 73.0%. In a study by Demetriou et al. [37], the authors observed identical cultures in 50% of the patients with neuroischaemic ulcers. Huang et al. [40] observed identical cultures in 80.0% of grade 2 wounds, with a marked decrease to 31.0% and 29.4% for grade 3 and grade 4 wounds, respectively. In the study by Nelson et al. [41], the authors found that pathogen results differed between swab and tissue biopsy in 58% of cases, with tissue biopsies detecting significantly more pathogens than swab cultures. Finally, Li et al. [43] reported identical cultures in 25% of cases and partial concordance (at least one matching microorganism) in 23.3% of the sample. Additionally, based on a visual assessment of microbial species distribution between swab and tissue biopsy specimens (Table 1), only three studies [32, 35, 36] reported identical ranking of microbial specimens, whereas rankings varied substantially in the remaining studies [28–31, 33, 34, 38–41, 43, 44].

**TABLE 1:** Characteristics of studies examining the diagnostic accuracy of swab vs. tissue biopsy for detecting infected diabetic foot wounds.

Author, year	Country	Sample	Number of samples collected	Swab collection technique	Tissue biopsy technique	Total number of pathogens and top three most prevalent
Pellizzer et al. 2001	Italy	*n* = 29 Age: mean of 58 years Males: 79.3% Duration of diabetes: mean of 14 years Wagner Grade > III: 100%	Swab: 29 Tissue biopsy: 29	Post‐debridement targeted swab	Post‐debridement punch biopsy (2 mm)	Swab: 68 *Staphylococcus aureus: 10* *Bacteroides spp: 9* *Enterococcus spp: 8* *Peptostreptococcus spp: 8* Tissue biopsy: 60 *Enterococcus spp: 12* *Staphylococcus aureus: 10* *Bacteroides spp: 6* *Streptococcus spp: 6* *Staphylococcus spp coagulase-negative: 6*

Kelkar & Kagal 2004	India	*n* = 50 Age: NR Males: NR Duration of diabetes: NR Wagner Grade > III: 100%	Swab: 50 Tissue biopsy: 50	Post‐debridement deep‐area swab	Post‐debridement homogenized tissue biopsy	Swab: 150 Tissue biopsy: 185

Slater et al. 2004	Israel	*n* = 56 Age: mean of 62.4 years Males: 64.3% Duration of diabetes: mean of 12.8 years Wagner Grade > III: 35.7%	Swab: 60 Tissue biopsy: 60	Pre‐debridement Z‐technique swab	Post‐debridement tissue biopsy	Swab: 161 *Staphylococcus aureus: 30* *Staphylococcus spp coagulase-negative: 21* *Streptococcus spp: 17* Tissue biopsy: 150 *Staphylococcus aureus: 30* *Staphylococcus spp coagulase-negative: 23* *Streptococcus spp: 15*

Mutluoglu et al. 2012	Turkey	*n* = 54 Age: mean of 62.5 years Males: 79.6% Duration of diabetes: mean of 15.5 years Wagner Grade > III: NR	Swab: 89 Tissue biopsy: 89	Pre‐debridement rotational swab	Post‐debridement excisional biopsy	Swab: 79 *Pseudomonas spp: 23* *Staphylococcus aureus: 17* *Enterococcus spp: 11* Tissue biopsy: 78 *Pseudomonas spp: 26* *Staphylococcus aureus: 17* *Enterococcus spp: 8*

Demetriou et al. 2013	Greece	*n* = 22 Age: mean of 75.1 years Males: 59.1% Duration of diabetes: mean of 17 years Wagner Grade > III: NR	Swab: 22 Tissue biopsy: 22	Post‐debridement swab using Amies transport	Post‐debridement punch biopsy	Swab: NR Tissue biopsy: NR
Dunyach‐Remy et al. 2014	France	*n* = 20 Age: above > 60 years Males: 75% Duration of diabetes: NR Wagner Grade > III: 65%	Swab: 20 Tissue biopsy: 20	Pre‐debridement Z‐technique swab	Post‐debridement deep tissue biopsy	Swab: 4 *Staphylococcus aureus: 2* *Staphylococcus epidermis: 1* *Streptococcus spp: 1* Tissue biopsy: 11 *Staphylococcus aureus: 8* *Streptococcus spp: 1* *Pseudomonas spp: 1* *Escherichia coli: 1*

Nelson et al. 2018	England	*n* = 395 Age: Mean of 63.1 years Males: 80% Duration of diabetes: Mean of 16.8 years Wagner Grade > III: 32.6%	Swab: 395 Tissue biopsy: 395	Post‐debridement Levine swab	Post‐debridement curette/scalpel biopsy	Swab: 264 *Staphylococcus aureus: 125* *Streptococcus spp: 48* *Staphylococcus spp methicillin-resistant: 27* Tissue biopsy: 379 *Staphylococcus aureus: 125* *Streptococcus spp: 61* *Enterococcus spp: 53*

Shahi et al. 2015	India	*n* = 42 Age: NR Males: NR Duration of diabetes: NR Wagner Grade > III: NR	Swab: 42 Tissue biopsy: 42	Pre‐debridement centre swab	Punch biopsy from ulcer base (6 mm)	Swab: 69 *Enterococcus spp: 14* *Alcaligenes spp: 8* *Staphylococcus aureus: 7* *Pseudomonas spp: 7* *Escherichia coli: 7* *Stenotrophomonas spp: 7* Tissue biopsy: 71 *Enterococcus spp: 10* *Escherichia coli: 9* *Pseudomonas spp: 8* *Alcaligenes spp: 8*

Huang et al. 2016	China	*n* = 56 Age: mean of 61.1 years Males: 62.5% Duration of diabetes: mean of 10 years Wagner Grade > III: 82.1%	Swab: 56 Tissue biopsy: 56	Post‐debridement Levine swab	Post‐debridement punch biopsy	Swab: 73 *Staphylococcus aureus: 15* *Streptococcus spp: 12* *Staphylococcus spp coagulase-negative: 9* Tissue biopsy: 74 *Staphylococcus aureus: 16* *Proteus: 11* *Enterococcus spp: 10*

Jouhar et al. 2020	Lebanon	*n* = 356 Age: mean of 66.9 years Males: 69.1% Duration of diabetes: mean of 18.4 years Wagner Grade > III: NR	Swab: 146 Tissue biopsy: 179	Surface swab	Surgical deep tissue biopsy	Swab: NR Tissue biopsy: NR

Attard et al. 2021	Malta	*n* = 20 Age: mean of 62 years Males: 70% Duration of diabetes: mean of 18 years Wagner Grade > III: 40%	Swab: 20 Tissue biopsy: 20	Post‐debridement Levine swab	Post‐debridement deep tissue biopsy	Swab: 21 *Staphylococcus aureus: 6* *Pseudomonas spp: 4* *Staphylococcus albus: 3* *Streptococcus spp: 3* Tissue biopsy: 28 *Staphylococcus aureus: 9* *Staphylococcus albus: 7* *Pseudomonas spp: 4*

Li et al. 2021	China	*n* = 60 Age: mean of 62.5 years Males: 75% Duration of diabetes: mean of 11.1 years Wagner Grade > III: NR	Swab: 60 Tissue biopsy: 60	Pre‐debridement Z‐technique swab	Surgical deep tissue biopsy	Swab: 61 *Staphylococcus aureus: 14* *Streptococcus spp: 11* *Proteus bacillus vulgaris: 9* Tissue biopsy: 53 *Staphylococcus aureus: 13* *Enterococcus spp: 12* *Proteus bacillus vulgaris: 9*

Williams 2007	US	*n* = 32 Age: NR Males: NR Duration of diabetes: NR Wagner Grade > III: NR	Swab: 128 Tissue biopsy: 32	Intraoperative deep‐contact swab	Intraoperative deep tissue biopsy	Swab: 52 *Staphylococcus spp methicillin-resistant: 8* *Streptococcus spp: 6* *Diphtheroids: 5* *Enterococcus spp: 5* *Pseudomonas spp: 5* Tissue biopsy: 52 *Staphylococcus spp methicillin-resistant: 9* *Pseudomonas spp: 7* *Streptococcus spp: 6*

Bozkurt et al. 2011	Turkey	*n* = 75 Age: mean of 57.3 years Males: 46.7% Duration of diabetes: mean of 9.2 years Wagner Grade III: 54.7%	Swab: 75 Tissue biopsy: 75	Pre‐debridement rotational swab	Post‐debridement curette biopsy	Swab: 81 *Streptococcus spp: 13* *Klebsiella spp: 11* *Escherichia coli: 10* Tissue biopsy: 70 *Klebsiella spp: 10* *Pseudomonas spp: 9* *Staphylococcus spp coagulase-negative: 8* *Streptococcus spp: 8* *Escherichia coli: 8* *Enterococcus spp: 8*

Daivasikamani et al. 2015	India	*n* = 112 Age: NR Males: NR Duration of diabetes: NR Wagner Grade > III: NR	Swab:112 Tissue biopsy:112	Pre‐debridement zigzag‐pressure swab	Punch biopsy from viable tissue	Swab: 96 *Staphylococcus spp: 31* *Escherichia coli: 13* *Commensals: 12* *Pseudomonas spp: 12* *Klebsiella spp: 12* Tissue biopsy: 116 *Staphylococcus spp: 37* *Escherichia coli: 18* *Klebsiella spp: 17*

Sankar et al. 2020	India	*n* = 77 Age: range of 40 to 81 years Males: 70.1% Duration of diabetes: above > 6 years Wagner Grade III: 67.5%	Swab: 77 Tissue biopsy: 77	Post‐debridement Levine swab	Post‐debridement punch biopsy (4 mm)	Swab: 68 *Staphylococcus aureus: 22* *Staphylococcus spp coagulase-negative: 17* *Pseudomonas spp: 9* *Klebsiella spp: 9* Tissue biopsy: 77 *Staphylococcus aureus: 29* *Staphylococcus spp coagulase-negative: 15* *Klebsiella spp: 11*

Kumar et al. 2023	India	*n* = 120 Age: mean of 56.9 years Males: 64.2% Duration of diabetes: NR Wagner Grade III: 60.8%	Swab: 120 Tissue biopsy: 120	Post‐debridement Levine swab	Post‐debridement punch biopsy (4 mm)	Swab: 125 *Staphylococcus aureus: 56* *Escherichia coli: 25* *Citrobacter spp: 13* Tissue biopsy:108 *Staphylococcus aureus: 61* *Escherichia coli: 15* *Citrobacter spp: 8*

### 3.3. Diagnostic Accuracy for Diabetic Foot Infections

Five studies [36, 37, 40–42] reported sufficient information on diagnostic accuracy outcomes. The diagnostic accuracy of swab cultures compared to tissue biopsy varied considerably. Mutluoglu et al. [36] reported a sensitivity and specificity of 79.4% and 52.4%, respectively, when assessing the accuracy of the culture results of the swab relative to tissue biopsy specimen. In the study by Demetriou et al. [37], swab cultures demonstrated a good sensitivity of 100% but a low specificity of 22.2% for detecting infection in patients with neuroischaemic ulcers. Huang et al. [40] observed that swab cultures had an overall sensitivity of 92% and specificity of 50% in identifying pathogens. When stratified by pathogen type, swab cultures performed better for Gram‐negative pathogens (sensitivity = 88.0%, specificity = 90.3%) than for Gram‐positive organisms (sensitivity = 90.6%, specificity = 79.2%). Other two studies [41, 42] had reported overall sensitivity and specificity as well as stratified by pathogen type. Nelson et al. [41] reported TP, FP, FN and TN values, with an overall sensitivity and specificity of 79.0% and 85.0% in identifying at least one pathogen, respectively. Stratified results for Gram‐positive organisms were 74.0% of sensitivity and 89.0% of specificity, and 63.0% of sensitivity and 95.0% of specificity for Gram‐negative organisms. Finally, Jouhar et al. [42] reported 94.0% sensitivity and 81.0% specificity for detecting Gram‐negative pathogens, and 76.0% sensitivity and 72.0% specificity for Gram‐positive pathogens.

**FIGURE 2:**
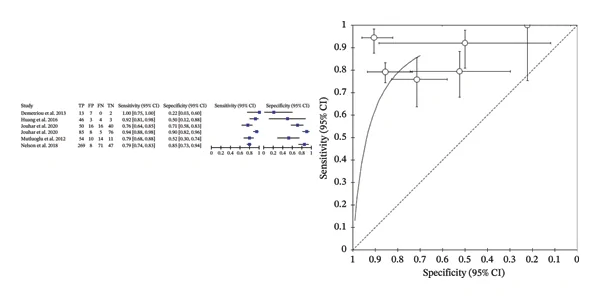
Random‐effects diagnostic accuracy meta‐analysis on the comparison between swab cultures and tissue biopsy in identifying diabetic foot infections. Horizontal lines represent the confidence intervals for specificity, while vertical lines indicate the 95% confidence intervals for the sensitivity of the individual studies.

In the meta‐analysis, the pooled sensitivity and specificity of swab cultures for detecting infected diabetic foot wounds were 84.8% (95% CI: 75.8%–90.8%) and 68.0% (95% CI: 45.6%–84.4%), respectively, using tissue biopsy as the reference standard (Figure 2). Heterogeneity, assessed through the Zhou and Dendukuri *I* ^2^ statistic, was 31.0%. The DOR was estimated at 11.8 (95% CI: 3.8–37.2), and the false‐positive rate was 32.0% (95% CI: 15.6%–54.4%). Positive and negative likelihood ratios were 2.65 (95% CI: 1.1–6.6) and 0.22 (95% CI: 0–10.0), respectively. Regarding the overall diagnostic performance, the AUC was 85.6% (95% CI: 83.1%–88.1%), with a normalised partial AUC of 85.7% (95% CI: 83.2%–88.2%). The SROC curve is presented in Figure 2. A publication bias analysis was not undertaken due to the small number of studies (*n* < 10).

**FIGURE 3:**
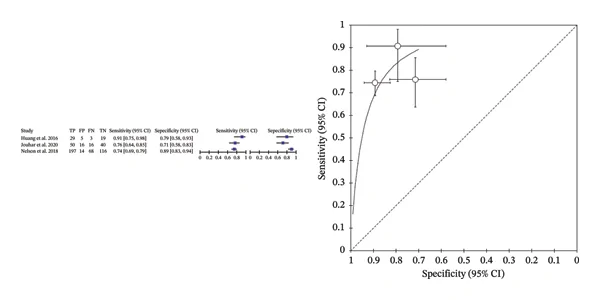
Random‐effects diagnostic accuracy meta‐analysis on the comparison between swab cultures and tissue biopsy in identifying Gram‐negative diabetic foot infections. Horizontal lines represent the confidence intervals for specificity, while vertical lines indicate the 95% confidence intervals for the sensitivity of the individual studies.

**FIGURE 4:**
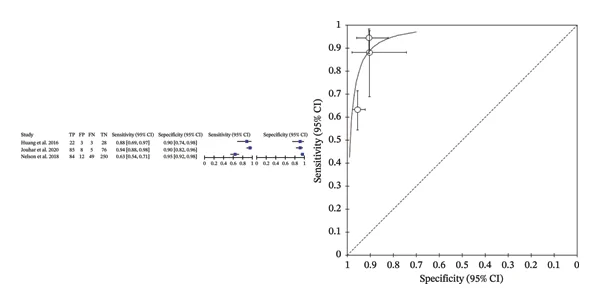
Random‐effects diagnostic accuracy meta‐analysis on the comparison between swab cultures and tissue biopsy in identifying Gram‐positive diabetic foot infections. Horizontal lines represent the confidence intervals for specificity, while vertical lines indicate the 95% confidence intervals for the sensitivity of the individual studies.

Subgroup analyses indicated better pooled sensitivity and specificity for Gram‐negative compared to Gram‐positive pathogens. For Gram‐negative pathogens, the pooled sensitivity and specificity of swab cultures compared to tissue biopsy were 85.2% (95% CI: 58.9%–95.6%) and 92.9% (95% CI: 87.3%–96.1%), respectively, with an I^2^ of 0%. The DOR was estimated at 74.6 (95% CI: 26.7–208.2), with positive and negative likelihood ratios of 12.0 (95% CI: 2.1–68.5) and 0.2 (95% CI: 0–1872.2), respectively. The AUC was 95.2% (95% CI: 93.3%–97.1%), with a normalized partial AUC of 81.7% (95% CI: 78.2%–85.3%). For Gram‐positive pathogens, swab cultures demonstrated a pooled sensitivity of 76.8% (95% CI: 71.4%–81.5%) and specificity of 81.0% (95% CI: 66.4%–90.2%), with an I^2^ of 0%. The DOR was estimated at 14.2 (95% CI: 7.1–28.3), with a positive likelihood ratio of 2.7 (95% CI: 2.0–8.4) and a negative likelihood ratio of 0.3 (95% CI: 0.1–10.0). The AUC was 80.3% (95% CI: 76.8%–83.8%), with a normalized partial AUC of 76.1% (95% CI: 72.3%–79.9%). Results are presented in Figures 3 and 4.

### 3.4. Additional Diabetic Foot Infection Outcomes

Only four studies [35, 39, 41, 42] reported sufficient information related to diabetic foot infections. Slater et al. [35] described severity‐based empirical antimicrobial therapy, which was adjusted according to deep tissue culture results in 10 cases. Among wounds with available follow‐up, 42 of 49 (85.7%) either healed or had infection resolution, although therapeutic outcomes were not the primary focus [35]. Shahi and Kumar [39] reported a high burden of antimicrobial resistance, with 38 of 142 isolates (26.8%) classified as multidrug resistant and class 1 integrons detected in 26 of these isolates. Several resistance‐associated genes were also identified, including β‐lactamase, methicillin‐resistant and vancomycin‐resistant genes. Nelson et al. [41] found moderate agreement between wound swab and tissue sample results regarding the need to change antimicrobial therapy (73.3%; kappa 0.45, 95% CI: 0.34–0.56), with tissue sampling more likely to indicate therapy change but also associated with more bleeding and pain. Finally, Jouhar et al. [42] reported that most hospitalised patients received intravenous antibiotics (93.0%), with a mean total antibiotic duration of 22 ± 16 days, and many also required vascular or surgical procedures.

## 4. Discussion

The present study aimed to synthesise current evidence comparing the diagnostic accuracy of swab cultures and tissue biopsies in infected diabetic foot wounds. We examined the concordance, sensitivity, specificity and overall diagnostic performance across the studies. Our findings indicate that swab cultures provide a pooled sensitivity and specificity above 85%, using tissue biopsy as the reference standard. Additionally, swab cultures showed a sensitivity of 76.8% and specificity of 81.0% for identifying Gram‐positive and even a sensitivity of 84.8% and specificity of 68.0% for any pathogen, despite variability and low concordance in microbial species across studies. Therefore, from a diagnostic perspective, this profile suggests that swab cultures may be more suitable for screening or initial assessment rather than for confirming infection or directing targeted antimicrobial therapy. Tissue biopsy is the gold standard and should be undertaken when adequate expertise and resources are available; however, swab cultures may add value to the clinical decision in resource‐limited settings or when biopsy is too invasive or contraindicated.

Two systematic reviews from 2006 [17, 18] included only three studies directly comparing swab cultures and tissue biopsy, concluding that the available evidence was insufficient. Despite widespread clinical use, swab cultures remain undervalued and neglected [16]. For example, in a qualitative study by Broom et al. [16], clinicians expressed a range of concerns regarding swab cultures, including gaps in training, diagnostic uncertainty and difficulties interpreting microbiological findings [16], all of which affect clinical decision‐making. This review provides the first quantitative comparison of swab vs. biopsy diagnostic performance and strengthens the evidence for informed practice.

Indeed, we observed a substantial heterogeneity in the identification of pathogens in diabetic foot infections. This variation may be explained by the range of cleansing or debridement steps undertaken before collection, with both pre‐ and post‐debridement undertaken in ∼50% of the cases, or antibiotic therapies, reported in ∼50% of the cases. Ulcer severity was another factor deemed important, with a previous study [40] reporting a concordance rate below 30% in grade 3 and 4 wounds. Available clinical guidelines [8–10] also indicated that swabs of open wounds are less sensitive and specific than tissue specimens, although based on limited evidence [33]. However, we found interesting results when evaluating the diagnostic accuracy of swab cultures vs. tissue biopsy for identifying Gram‐negative pathogens. The specific sensitivity of 85%, specificity of 93% and AUC of 95% were unexpected within the context of diabetic foot infections, although the lack of sufficient studies included in our meta‐analysis. Additionally, the positive likelihood ratio of 12.0 indicates that a positive swab result is relatively reliable for confirming these infections, while the negative likelihood ratio of 0.2 indicates an interesting ability to rule them out, despite the limited number of studies, which resulted in imprecise CIs. Our finding warrants future studies examining the role of swab cultures in this setting, focusing on Gram‐negative pathogens and clinical scenarios where tissue biopsy may not be feasible. We suggest that swab cultures may be considered once Gram‐negative pathogens are identified, particularly *Pseudomonas* spp., *Escherichia coli*, and *Proteus* spp., to help in the selection of the most appropriate antimicrobial therapy, provided that specimens are collected using standardised, post‐debridement techniques, as recommended by the IWGDF guideline [8, 10]. Nevertheless, in cases where a Gram‐negative pathogen is isolated by swab, clinicians should carefully consider the possibility of polymicrobial infection and interpret the findings within the clinical context.

Swab cultures identified Gram‐positive infections in ∼77% of cases and ruled them out in 81%. Among potential pathogens, *Staphylococcus aureus* is one of the most prevalent Gram‐positive aerobic organisms in diabetic foot infections, accounting for ∼24% of all isolates [45]. Given the diagnostic accuracy observed, the identification of Gram‐positive should not be solely used to narrow antimicrobial coverage. Once a resistant Gram‐positive organism is identified, this finding must be carefully considered for treatment planning along with clinical signs and other microbiological or inflammatory markers.

For overall diagnostic performance, swab cultures provided sensitivity of 84.8% and specificity of 68.0% for any pathogen. Conversely, the false‐positive rate of 32% suggests that swab cultures may also reflect colonising or commensal bacteria, potentially leading to unnecessary use of broad‐spectrum antimicrobials. This limitation may be particularly relevant in pre‐debridement samples, as diabetic foot ulcers often contain ischaemic or necrotic tissue with bacterial biofilm. Nevertheless, this is specifically relevant in the case of polymicrobial infections, which are common among patients who have undergone previous antibiotic treatment [46]. In such cases, swab cultures can still identify many pathogens, assisting with the first‐line assessment during primary care or outpatient settings. This is particularly important where biopsy is contraindicated (e.g., ischemic tissue, surgical contraindication), access to tissue biopsy may be limited (e.g., mild or superficial infections) and rapid clinical decision‐making is needed. However, it is important to note that isolations of anaerobic pathogens, common in ∼17% of deep diabetic foot infections [47], were not frequent in the included studies. There are limitations and difficulties in the isolation of anaerobic pathogens through swab cultures, which affected our ability to account for the potential impact of anaerobic pathogens. These should still be considered in the treatment of deeper or more complex infections.

This is the first systematic review and meta‐analysis comparing the diagnostic accuracy of swab cultures and tissue biopsies in patients with infected diabetic foot wounds. The strengths of this review include (i) compiling data from 17 studies with 1581 participants, making it the largest synthesis on this topic; (ii) performing a meta‐analysis for the first time to investigate the diagnostic accuracy of swab cultures in diabetic foot infections and (iii) conducting subgroup analysis for Gram‐positive and Gram‐negative organisms to explore performance by pathogen type. Nevertheless, our study has limitations. First, most included studies were conducted in Asia (64.7%), which may limit the generalisability of our findings to other regions with different healthcare systems or microbiological profiles, as previously reported [45]. This potential geographic bias warrants future research. Second, the lack of standardisation in swab collection techniques across studies represents an important source of bias. Variations in sampling methods, including pre‐ versus post‐debridement collection and the use of different techniques (e.g., Levine versus Z‐technique), may have significantly influenced microbiological and diagnostic accuracy estimates. Additionally, the timing of swab collection in relation to wound debridement represents an additional source of variability across studies. Insufficient reporting prevented subgroup analyses comparing pre‐ and post‐debridement swab sampling, which may have influenced microbiological yield and diagnostic accuracy. This methodological heterogeneity limits the comparability of studies and may partially explain the variability observed in our findings. Third, the reporting quality was very low, with just a few studies providing sufficient information to be included in the meta‐analysis. Fourth, non‐English language studies were not included. Finally, publication bias was not tested given the small number of studies (*n* = 5). Overall, future studies should prioritise the use of standardised, guideline‐recommended swab collection techniques to allow more accurate comparisons with tissue biopsy and improve the reliability of diagnostic accuracy estimates.

In summary, our findings are that swab cultures could identify Gram‐negative pathogens and may still be acceptable for detecting Gram‐positive and overall infections. While tissue biopsy remains the reference standard, swab cultures may represent a practical and accessible complementary tool in settings where tissue biopsy is not feasible or not routinely performed. These findings should be interpreted with caution and warrant further research to determine their role across different clinical scenarios.

## Author Contributions

Conception and design: Roberto S. Heck, ⁠Rodrigo S. Lins, ⁠Pedro Lopez and ⁠Lessandra Michelin; acquisition, analysis or interpretation of data: Roberto S. Heck, ⁠Rodrigo S. Lins, Daniela R. Speransa, ⁠Pedro Lopez and ⁠Lessandra Michelin; drafting of the manuscript: Roberto S. Heck, ⁠Rodrigo S. Lins, ⁠Daniela R. Speransa, Pedro Lopez and ⁠Lessandra Michelin; critical revision of the manuscript for important intellectual content: Roberto S. Heck, ⁠Rodrigo S. Lins, Daniela R. Speransa, ⁠Pedro Lopez and ⁠Lessandra Michelin.

## Funding

No financial support was received to conduct the present study or for the preparation or publication of this manuscript.

## Disclosure

Sponsors were not involved in the study design, analysis or interpretation of data, manuscript writing and decision to submit the manuscript for publication. All authors read and approved the final version of the article.

## Consent

This study is a systematic review and did not involve direct interaction with human subjects or the collection of individual patient data. Therefore, patient consent was not required. The study was conducted in accordance with the ethical standards applicable to secondary analyses and complies with the current regulations in Brazil.

## Conflicts of Interest

The authors declare no conflicts of interest.

## Supporting Information

Additional supporting information can be found online in the Supporting Information section.

## Supporting information


**Supporting Information** Appendix S1. Search strategy. Table S1. Individual risk of bias assessment.

## Data Availability

Data used in the present study such as data extraction templates, forms and analysis will be made available upon reasonable request to the corresponding author.
