# Supplementary material for: A Systematic Review and Meta‐Analysis Comparing the Diagnostic Accuracy of Swab Cultures and Tissue Biopsy for Diagnosing Diabetic Foot Infections
Source: Int J Endocrinol. 2026 Aug 20;2026:6364693. doi: 10.1155/ije/6364693 (PMC13494358; doi:10.1155/ije/6364693)
Supplement: Supplementary file 1 [file IJE-2026-6364693-s001.docx]

**A SYSTEMATIC REVIEW AND META-ANALYSIS COMPARING THE DIAGNOSTIC ACCURACY OF SWAB CULTURES VS. TISSUE BIOPSY FOR DIAGNOSING DIABETIC FOOT INFECTIONS**

**Appendix S1.** Search strategy.

**PubMed search**

("Diabetic Foot" [Title/Abstract] OR "Foot, Diabetic" [Title/Abstract] OR "Diabetic Feet" [Title/Abstract] OR "Feet, Diabetic" [Title/Abstract] OR "Foot Ulcer, Diabetic" [Title/Abstract] OR "Diabetic Ulcer" [Title/Abstract] OR "Diabetic Wound" [Title/Abstract] OR "Diabetic Foot Ulcer" [Title/Abstract]) AND ("swab" [Title/Abstract] OR "swab culture" [Title/Abstract] OR swabbing [Title/Abstract] OR "wound swab" [Title/Abstract] OR "superficial swab" [Title/Abstract] OR "tissue biopsy" [Title/Abstract] OR "deep tissue sample" [Title/Abstract])

**CINAHL and Web of Science search**

("Diabetic Foot" OR "Foot, Diabetic" OR "Diabetic Feet" OR "Feet, Diabetic" OR "Foot Ulcer, Diabetic" OR "Diabetic Ulcer" OR "Diabetic Wound" OR "Diabetic Foot Ulcer") AND ("swab" OR "swab culture" OR "wound swab" OR "superficial swab" OR "tissue biopsy" OR "deep tissue sample")

**Embase search**

('diabetic foot':ab,ti OR 'diabetic foot ulcer':ab,ti OR 'diabetic wound':ab,ti) AND ('swab':ab,ti OR 'swabbing':ab,ti OR 'biopsy':ab,ti OR 'biopsy technique':ab,ti)

**Table S1.** Individual risk of bias assessment.

| **Study** | **Patient Selection** | **Index Test** | **Reference Standard** | **Flow and Timing** |
| --- | --- | --- | --- | --- |
| **Jouhar et al. (2020)** | Low | Low | Low | Unclear |
| **Pellizzer et al. (2001)** | Low | Low | Low | Low |
| **Kelkar et al. (2004)** | High | Unclear | Low | Low |
| **Slater et al. (2004)** | Low | Low | Low | Low |
| **Mutluoglu et al. (2012)** | Unclear | Low | Low | Low |
| **Demetriou et al. (2013)** | High | Unclear | Low | Unclear |
| **Dunyach-Remy et al. (2014)** | Low | Low | Low | Low |
| **Shahi et al. (2015)** | Unclear | Unclear | Low | Unclear |
| **Huang et al. (2016)** | Low | Low | Low | Low |
| **Nelson et al. (2018)** | Low | Low | Low | Low |
| **Li et al. (2021)** | Low | Low | Low | Unclear |
| **Camilleri Attard et al. (2021)** | Low | Low | Low | Unclear |
| **Kumar et al. (2023)** | Low | Low | Low | Low |
| **Williams et al. (2007)** | High | Low | Low | Low |
| **Bozkurt et al. (2011)** | Low | Low | Low | Low |
| **Daivasikamani et al. (2015)** | Unclear | Low | Low | Low |
| **Sankar et al. (2020)** | Low | Low | Low | Low |
